# ASSOCIATION BETWEEN THE NIH TOOLBOX COGNITION BATTERY AND GAIT IN OLDER ADULTS

**DOI:** 10.1093/geroni/igz038.3388

**Published:** 2019-11-08

**Authors:** Kate Hamel, Sarah Barber, Carl Ketcham, Kristy Lui

**Affiliations:** 1 San Francisco State University, San Francisco, California, United States; 2 Georgia State University, Atlanta, Georgia, United States

## Abstract

The relationship between older adults’ gait and cognition has been well-studied, however there is little consensus regarding a best set of measures to assess cognition. One option that has not been previously examined is the NIH Toolbox Cognition Battery (NIHT-CB), which was developed to be used across the lifespan and across research disciplines. This study examined the relationships between the seven subtests of the NIHT-CB, Trail-Making Tests A and B, and temporospatial measures of gait. One hundred sixty-seven healthy, community-dwelling older adults (115 females, 73.4 ± 4.5 years) completed these cognitive measures and also walked at their self-selected pace back-and-forth five times along a temporospatial-measuring walkway. The mean and coefficient of variation were calculated for each gait variable (stride length, width, time and velocity; stance/swing time and % of stride). After controlling for potential confounders (height, weight, age, sex, education, self-efficacy, health, exercise and falls history), executive function measures were typically the most significant cognitive predictors. More specifically, the Dimensional Change Card Sort task was the best predictor of temporal measures and stride velocity (all ps < 0.001) and the Trail-Making Test Part B was the best predictor for variability measures (five of eight p-values < 0.001). Interestingly, stride length and also stance and swing % of stride were strongly related to a measure of language - Picture Vocabulary (all ps < 0.006). The NIHT-CB appears to be a useful tool for studies of gait in older adults, particularly when used in conjunction with Trail-Making Test B.

